# Application of the COOP/WONCA charts to aged patients with chronic obstructive pulmonary disease: a comparison between Japanese and Chinese populations

**DOI:** 10.1186/1471-2458-13-754

**Published:** 2013-08-15

**Authors:** Midori Yamaguchi, Motoyuki Nakao, Hideto Obata, Hideki Ikeda, Tetsuro Kanda, Qiao Wang, Yoriko Hara, Hisamitsu Omori, Yoko Ishihara

**Affiliations:** 1 Department of Public Health, School of Medicine, Kurume University, Kurume, Fukuoka 830-0011, Japan; 2 Yamaguchi-Ken Saiseikai Shimonoseki General Hospital, Yamaguchi 759-6603, Japan; 3 Nagasaki Goto Chuoh Hospital, Nagasaki 853-0031, Japan; 4 Institute of Population and Labor Economics, Chinese Academy of Social Sciences, Beijing 100732, China; 5 Department of Public Health, Faculty of Life Sciences, Kumamoto University, Kumamoto 860-8556, Japan

**Keywords:** COOP/WONCA chart, SF-36, Chronic pulmonary diseases, Health status, Asians, Japanese, Chinese

## Abstract

**Background:**

The prevalence of chronic obstructive pulmonary disease (COPD) is similar in Japan and China and is increasing due to high rates of smoking in these countries. Reducing COPD is an important public health issue. The goals of this study were to verify the reliability and validity of the Japanese version of the COOP/WONCA charts, a tool for measuring health status, and to examine the qualitative differences in health status between Japanese and Chinese patients with COPD and between these patients and healthy subjects.

**Methods:**

From 2008 to 2011, we examined the factors affecting the health status of Japanese and Chinese populations living in six cities. Participants were patients with COPD staged according to the Global Initiative for Chronic Obstructive Lung Disease (GOLD) criteria (140 Japanese, 201 Chinese) and healthy subjects (243 Japanese, 199 Chinese), all 50 to 79 years old. Health status was measured by using the COOP/WONCA charts, and basic information such as smoking status and medical history was reported by the participants.

**Results:**

The Japanese and Chinese versions of the COOP/WONCA charts were shown to be reliable and valid by test-retest, comparison with the SF-36 and respiratory symptoms, and correlation of results obtained from patients and their physicians. Stepwise multiple regression analyses demonstrated that “Physical fitness”, “Daily activities”, and “Social activities” were predicted by COPD status and/or respiratory symptoms; “Feelings” by nationality and respiratory symptoms; “Pain” by sex and respiratory symptoms; and “Overall health” by nationality. When the COOP/WONCA scores were stratified by nationality, age, sex, and COPD status, the difference of each score between the patients and healthy subjects was larger for the Chinese subjects than for the Japanese. The physical, psychosocial activities, and pain scores increased significantly as COPD status worsened in Chinese subjects, whereas these scores were not affected by sex, age, or COPD status for Japanese subjects. Brinkman index and use of smoky fuel indoors affected the COOP/WONCA scores in Chinese patients but not in Japanese patients.

**Conclusions:**

The Japanese COOP/WONCA charts are reliable and valid. COPD more severely affected the health status of Chinese participants than of Japanese participants. These results suggest that countermeasures against insufficient health care and smoky environments may improve the health status of Chinese patients with COPD.

## Background

Chronic obstructive pulmonary disease (COPD) is the fourth leading cause of death in the world. The main risk factors for COPD are cigarette smoking [1] and aging [2], and its prevalence and related mortality are expected to increase as the population ages [3]. Therefore, development of a strategy to prevent and treat COPD is an urgent issue in public health. Because the increased numbers of COPD patients represent an economic and social burden [4], there is a need for countermeasures in Asia where the air pollution is severe [5], the population is aging rapidly, and smoking rates are high.

Smoking rates are higher in Asia than in the Western developed countries. The smoking rate in Japan is 23.4% (38.2% for men and 10.9% for women) [6], although it has declined rapidly in response to a governmental campaign [7]. According to the World Health Organization [6], the smoking rate in China is 26.1% (52.1% for men and 2.3% for women). Similarly, a study of the general population of China aged 15 years and older reported the smoking rate to be 27.7% (52.1% for men and 2.3% for women) [8]. The prevalence of COPD is 10.9% (16.4% for men and 5.0% for women) in Japan [9] and 8.2% (12.4% for men and 5.1% for women) in China [10]. Another study reported a prevalence of 9.3% for men and 5.1% for women among 602 subjects from the general population of Guangzhou, China [11].

The smoking rate is currently decreasing in Japan, whereas in China the smoking rate is increasing. The prevalence of COPD and mortality from the disease are expected to increase due to prolonged life expectancy. The economic burden of treatment for COPD [12] and the social burden of the loss of disability-adjusted life years due to COPD [13] are also increasing because the prevalence of COPD, which is a slowly progressive chronic disease and puts patients at increased risk of infectious disease [14], is greatly elevated in those who are 40 years or older. However, little is known about the health status of older patients with COPD in Asia, and no study has compared the health status of Japanese and Chinese populations, who live with completely different medical systems, environments, and other patient circumstances.

International comparative studies using questionnaires must consider the cultural background of participants, such as language and customs. In addition, for research targeting a geriatric subject, a simplified questionnaire is required that is written in short, easy-to-understand sentences; is visually apparent; and can be completed in a short time. The COOP/WONCA charts, which consist of seven simple questions with associated drawings, were developed by the World Organization of National Colleges, Academies and Academic Associations of General Practitioners/Family Physicians (WONCA) to assess the health status of patients visiting primary care physicians. The reliability and validity of the English [15] and Chinese [16] versions have been verified. However, the Japanese version of the COOP/WONCA charts has been examined only for schoolchildren and not for adults [17-19]. Only a few studies have examined the health status of patients with COPD by using the COOP/WONCA charts in Western countries and China, and few studies have evaluated the health status of older patients with COPD, although one international comparative study has used the COOP/WONCA charts [20]. Therefore, we performed this study to verify the reliability and validity of the Japanese version of the COOP/WONCA charts and to examine the qualitative differences of health status between Japanese and Chinese patients with COPD ages 50-79 years and between these patients and healthy subjects.

## Methods

### Subjects

The survey was conducted in six cities in Japan and China from 2008 to 2011. The sites are Shimonoseki-shi (Yamaguchi prefecture), Goto-shi (Nagasaki prefecture), and Kumamoto-shi (Kumamoto prefecture) in southwestern Japan and Beijing, Shanghai, and Ulanhot (Inner Mongolia) in China. Patients ages 50-79 years visiting outpatient clinics and healthy subjects having medical checkups at health care centers were enrolled. This study was approved in advance by the Clinical Ethics Committee of Kurume University, Fukuoka, Japan. The objectives and other details of the study were explained to the subjects, and their written informed consent was obtained.

Pulmonologists examined the pulmonary function of the patients with COPD in hospitals or clinics, diagnosed respiratory disease, and determined COPD stage (I, II, III, or IV) using the Global Initiative for Chronic Obstructive Lung Disease (GOLD) criteria [21]. Patients with home oxygen therapy were excluded. Pulmonary function of healthy subjects was tested in health care centers. Healthy subjects did not have dyslipidemia or respiratory, circulatory, gastrointestinal, or endocrine disease.

The questionnaire is a self-administered booklet that consists of requests for basic information, four questions related to respiratory symptoms (“cough caused by the weather”, “sputum without infectious disease”, “sputum in the morning”, and “wheezing”), and the COOP/WONCA charts. Medical staff distributed and collected the questionnaires at the hospitals and clinics. Health care centers mailed the questionnaires to healthy participants beforehand and collected them at health checkups. Permission to use the Japanese version of SF-36v2 (SF-36) Additional file 1 was obtained from Quality Metric (New York, NY, USA). For the Japanese and Chinese versions of the COOP/WONCA charts Additional file 2, permission was obtained from the Japan Primary Care Association and Dr. Cindy Lam, who developed the Chinese version of the COOP/WONCA charts. The responses to the COOP/WONCA charts are scored on a five-point ordinal scale ranging from 1 to 5 (1 = best, 5 = worst). The responses to the SF-36 are on three-, five-, or six-point ordinal scales, from which we calculated the eight subscales from 0 to 100 points (max = 100) as instructed in the manual [22].

### Analytical methods

All completed questionnaires were assigned identification numbers after collection. All cases missing values in the questionnaire were removed from the data set before the statistical analyses were performed. Data were analyzed by *t*-test, chi-square test, correlation analysis, and a multiple-regression analysis by using JMP Ver.10.0 (SAS Institute, Cary, NC, USA) and SPSS Ver.17 (IBM, Armonk, NY, USA). Probability less than 0.005 was considered to be statistically significant.

## Results

### Characteristics of participants

Study subjects included 140 COPD patients with GOLD status I-IV in Japan and 201 in China, as well as 243 healthy subjects in Japan and 199 in China (Table 1). The ratio of men to women was 3:2 for the Japanese and 1:1 for the Chinese group. Approximately 40% of the males of both nationalities had white-collar jobs, and approximately 27% (Japanese) and 37% (Chinese) had blue-collar jobs. Approximately 33% of Japanese men and 20% of Chinese men were retired, as was expected because this study was targeted to older individuals. The proportion of housewives was high among both population groups. In addition, Chinese women were more likely to have blue-collar jobs than were Japanese women.

**Table 1:** Characteristics of participants

		**Male (n = 438)**			**Female (n = 345)**		
				**Chi-squared**			**Chi-squared**
		**Japanese**	**Chinese**		**Japanese**	**Chinese**	
Population (n (%))		230 (52.5)	208 (47.5)		153 (44.3)	192 (55.7)	
Age (y)	(mean ± SD)	62.77 ± 8.53	65.84 ± 7.69		60.10 ± 7.62	63.86 ± 8.46	
Body Mass Index	(mean ± SD)	23.06 ± 3.30	23.61 ± 3.86		22.37 ± 3.06	24.02 ± 3.88	
Job (n (%))	White collar	92 (51.4)	87 (48.6)	0.0056	50 (50.0)	50 (50.0)	0.0245
	Blue collar	63 (45.3)	76 (54.7)		33 (33.0)	67 (67.0)	
	Housewife	0 (−)	0 (−)		70 (48.3)	75 (51.7)	
	Retired	75 (64.1)	42 (35.9)		0 (−)	0 (−)	
	Missing	0 (0.0)	3 (100.0)		0 (−)	0 (−)	
Group (n (%))	Healthy	133 (63.0)	78 (37.0)	< 0.0001	110 (47.6)	121 (52.4)	0.0817
	COPD	97 (42.7)	130 (57.3)		43 (37.7)	71 (62.3)	
Cigarette smoking (n (%))	Healthy subjects						
	Current smoker	43 (66.2)	22 (33.8)	< 0.0001	9 (75.0)	3 (25.0)	0.0045
	Ex-smoker	61 (87.1)	9 (12.9)		5 (100.0)	0 (0.0)	
	Non smoker	29 (43.3)	38 (56.7)		96 (45.7)	114 (54.3)	
	Missing	0 (0.0)	9 (100.0)		0 (0.0)	4 (100.0)	
	COPD patients						
	Current smoker	13 (28.3)	33 (71.7)	0.0007	2 (25.0)	6 (75.0)	0.0041
	Ex-smoker	67 (52.3)	61 (47.7)		6 (22.2)	21 (77.8)	
	Non smoker	17 (40.5)	25 (59.5)		35 (50.0)	35 (50.0)	
	Missing	0 (0.0)	11 (100.0)		0 (0.0)	9 (100.0)	
Brinkman Index (n (%))	Healthy subjects						
	0	29 (43.3)	38 (56.7)	< 0.0001	96 (45.7)	114 (54.3)	0.0064
	0 < BI 500	44 (64.7)	24 (35.3)		13 (81.3)	3 (18.8)	
	BI > 500	60 (89.6)	7 (10.4)		1 (100.0)	0 (0.0)	
	Missing	0 (0.0)	9 (100.0)		0 (0.0)	4 (100.0)	
	COPD patients						
	0	17 (40.5)	25 (59.5)	< 0.0001	35 (50.0)	35 (50.0)	0.0036
	0 < BI 500	17 (27.9)	44 (72.1)		4 (28.6)	10 (71.4)	
	BI > 500	63 (55.8)	50 (44.2)		4 (19.0)	17 (81.0)	
	Missing	0 (0.0)	11 (100.0)		0 (0.0)	9 (100.0)	
Heating sources (n (%))	Biomass, Coal, Animal dung	0 (0.0)	52 (100.0)	< 0.0001	0 (0.0)	41 (100.0)	< 0.0001
	Gas, Electric, Steam	230 (60.1)	153 (39.9)		153 (50.7)	149 (49.3)	
	Missing	0 (0.0)	3 (100.0)		0 (0.0)	2 (100.0)	

COPD status was determined in accordance with the GOLD criteria. Among male participants, 42.2% of Japanese and 62.5% of Chinese were patients with COPD; among female participants, 28.1% of Japanese and 37.0% of Chinese were patients with COPD (Table 1). The percentages of current and ex-smokers among healthy subjects was 78.2% of Japanese men, 39.7% of Chinese men, 12.7% of Japanese women, and 2.5% of Chinese women. Among the healthy subjects, more Japanese than Chinese were current or ex-smokers, regardless of sex. A higher proportion of Japanese than of Chinese male patients had both smoking experience and COPD; however, the proportion of female COPD patients with smoking experience was higher in the Chinese than in the Japanese. A higher percentage of patients with COPD than of healthy subjects had a Brinkman Index (BI; number of cigarettes smoked per day times smoking years) greater than 500. All Japanese participants used gas or electricity for cooking and heating, whereas 21%-25% of Chinese participants used fuel that causes indoor air pollution, such as coal, which was the most common heating source. Some participants in Ulanhot, Inner Mongolia, used wood, the dried dung of sheep, or dried corncobs as heating sources.

### The reliability and validity of the Japanese and Chinese versions of the COOP/WONCA charts

We examined the correlation between COOP/WONCA chart scores and SF-36 subscale scores in matched subjects (same sex and age). For participants of both nationalities, the “Physical fitness”, “Social activities”, “Overall health”, and “Pain” COOP/WONCA charts were highly correlated with the SF-36 subscales “Physical functioning”, “Role emotional”, “General health perceptions”, and “Bodily pain,” respectively (Table 2). The “Feelings” and “Daily activities” COOP/WONCA chart items correlated with different subscales of SF-36, depending on nationality. In the Japanese group, the COOP/WONCA scores of “Feelings” and “Daily activities” were correlated with the scores of “Mental health” and “Vitality” of the SF-36 subscales. In contrast, in the Chinese group, the COOP/WONCA scores of “Feelings” and “Daily activities” showed the highest correlation with “General health perceptions” and “Bodily pain” of the SF-36 subscales. In both groups, the correlations between the COOP/WONCA charts and the related subscales of the SF-36 were relatively high except for “Change in health”. Test-retest reliability was examined with 104 Japanese and 22 Chinese participants, with a one-month interval between tests. The intraclass correlation coefficients (ICC) ranged from 0.696 to 0.890 in the Japanese subjects and from 0.657 to 0.817 in the Chinese subjects, except for “Change in health” (data not shown), demonstrating that both the Japanese and Chinese versions of the COOP/WONCA charts had excellent reliability.

**Table 2:** Correlation between the COOP/WONCA charts and SF-36 subscales in age and sex matched Japanese and Chinese subjects

**Japanese (n = 26)**	**COOP/WONCA chart**						
	**Physical fitness**	**Feelings**	**Daily activities**	**Social activities**	**Change in health**	**Overall health**	**Pain**
SF-36 subscale							
Physical functioning	−0.508	−0.180	−0.344	−0.505	−0.312	−0.151	−0.424
Role physical	−0.368	−0.046	−0.262	−0.569	−0.031	−0.288	−0.237
Bodily pain	−0.176	−0.141	−0.184	−0.074	−0.287	−0.278	−0.797
General health perceptions	−0.196	−0.235	−0.367	−0.497	−0.022	−0.472	−0.266
Vitality	−0.360	−0.608	−0.632	−0.510	−0.102	−0.241	−0.441
Social functioning	−0.492	−0.484	−0.597	−0.668	−0.000	−0.397	−0.238
Role emotional	−0.392	−0.191	−0.332	−0.688	−0.159	−0.263	−0.258
Mental health	−0.304	−0.674	−0.454	−0.545	−0.192	−0.238	−0.465
**Chinese (n = 26)**	**COOP/WONCA chart**						
	**Physical fitness**	**Feelings**	**Daily activities**	**Social activities**	**Change in health**	**Overall health**	**Pain**
SF-36 subscale							
Physical functioning	−0.847	−0.872	−0.793	−0.758	−0.097	−0.676	−0.866
Role physical	−0.695	−0.831	−0.789	−0.780	−0.316	−0.675	−0.883
Bodily pain	−0.617	−0.875	−0.882	−0.843	−0.456	−0.656	−0.905
General health perceptions	−0.773	−0.912	−0.876	−0.848	−0.283	−0.720	−0.899
Vitality	−0.528	−0.735	−0.605	−0.632	−0.014	−0.537	−0.745
Social functioning	−0.469	−0.741	−0.739	−0.759	−0.333	−0.487	−0.726
Role emotional	−0.654	−0.826	−0.859	−0.869	−0.449	−0.712	−0.903
Mental health	−0.414	−0.673	−0.591	−0.638	−0.170	−0.428	−0.672

Data are presented as Pearson’s correlation coefficients.

Next, to assess the external validity of the COOP/WONCA chart, we examined the correlation of COOP/WONCA chart scores between Japanese and Chinese patients with COPD and their physicians, matched with age, sex, and COPD status. The scores for all of the items, except for “Pain” in the Japanese group, were highly correlated between the physicians and the patients (Table 3). For both nationalities, the scores assigned by the physicians were higher (worse) than those assigned by the patients, except for “Pain” in the Japanese group and “Daily activities” in the Chinese group. The difference of the “Pain” score between the physicians and patients was relatively large (mean, –0.46) in the Japanese population, indicating that the patients felt more severe pain than their physicians recognized.

**Table 3:** Correlation of COOP/WONCA chart scores assigned by the patients and their physicians in age and sex matched Japanese and Chinese patients with COPD

	**Pearson’s correlation coefficient**	**Physician’s score**	**Patient’s score**
Japanese (n = 13)			
Physical fitness	0.801	4.23 ± 0.73	4.00 ± 1.29
Feelings	0.617	1.85 ± 0.80	1.54 ± 0.52
Daily activities	0.722	2.15 ± 0.99	1.92 ± 0.95
Social activities	0.527	1.23 ± 0.44	1.08 ± 0.28
Change in health	0.815	3.15 ± 0.38	3.00 ± 0.82
Overall health	0.850	3.00 ± 0.71	2.77 ± 0.83
Pain	0.237	1.46 ± 0.78	1.92 ± 1.12
Chinese (n = 13)			
Physical fitness	0.965	3.85 ± 0.90	3.77 ± 1.01
Feelings	0.941	2.00 ± 0.82	1.92 ± 0.76
Daily activities	0.974	2.54 ± 1.20	2.62 ± 1.12
Social activities	0.953	2.00 ± 0.91	1.92 ± 0.86
Change in health	0.980	3.31 ± 1.38	3.23 ± 1.30
Overall health	0.826	3.92 ± 0.95	3.77 ± 0.93
Pain	0.978	2.38 ± 1.26	2.31 ± 1.32

Data in scores and differences are presented as mean ± SD.

We also examined the correlation between COOP/WONCA chart scores and single predictive variables (age, sex, body mass index, BI, heating sources, the number of respiratory symptoms, and COPD status). For the Japanese group, “Physical fitness”, “Feelings”, and “Social activities” were highly correlated with age, and five items (“Physical fitness”, “Feelings”, “Social activities”, “Change in health”, and “Overall health”) were correlated with the number of respiratory symptoms and COPD status (Table 4, upper panel). For the Chinese group, “Physical fitness” and “Overall health” were correlated with age, the rest of the scores except for “Change in health” were correlated with both BI and the number of respiratory symptoms, and all scores were highly correlated with COPD status (Table 4, lower panel). Moreover, “Daily activities” and “Social activities” were correlated significantly with heating sources.

**Table 4:** Spearman’s rank correlation coefficient between COOP/WONCA chart scores and subjects’ parameters

	**COOP/WONCA chart**						
**Japanese (n = 370)**	**Physical fitness**	**Feelings**	**Daily activities**	**Social activities**	**Change in health**	**Overall health**	**Pain**
Age	0.244**	−0.175*	0.021	−0.203**	−0.120	−0.043	−0.108
Sex	0.093	0.065	−0.045	0.029	−0.089	−0.101	0.173*
Body Mass Index	0.007	−0.007	−0.064	−0.023	−0.026	−0.084	0.003
Brinkman index	−0.031	−0.036	0.030	−0.057	0.015	0.141	−0.112
Heating sources	-	-	-	-	-	-	-
Number of respiratory symptoms	0.175*	−0.010	0.095	−0.009	−0.047	0.105*	0.089
COPD status	0.213**	−0.202**	0.011	−0.191**	−0.148*	0.018	−0.037
	**COOP/WONCA chart**						
**Chinese (n = 400)**	**Physical fitness**	**Feelings**	**Daily activities**	**Social activities**	**Change in health**	**Overall health**	**Pain**
Age	0.228**	0.069	0.066	0.104	0.073	0.174*	0.088
Sex	−0.009	−0.020	−0.091	−0.106	0.026	−0.036	0.088
Body Mass Index	−0.070	−0.034	−0.086	−0.055	0.041	−0.130	−0.041
Brinkman index	0.257**	0.308**	0.396**	0.328**	−0.030	0.253**	0.206**
Heating sources	0.009	0.100	0.157*	0.146*	−0.132	0.023	0.139
Number of respiratory symptoms	0.368**	0.547**	0.653**	0.616**	0.098	0.398**	0.407**
COPD status	0.452**	0.575**	0.738**	0.681**	0.200**	0.484**	0.429**

*: *P*<0.005, **: *P<*0.001.

### Stepwise multiple regression analyses to determine the predictive variables affecting each item of the health status

To determine which variables predict the COOP/WONCA chart scores, we performed stepwise multiple regression analyses with the scores of each COOP/WONCA chart item as dependent variables and population group, sex, age, COPD status, the number of respiratory symptoms, BI, and heating sources as predictive variables. The variance inflation factors were 2 or less, and the all F-values resulting from the analyses of variance were significant at *P* < 0.0001. The predictive variables with standardized partial regression coefficients > 0.2 were COPD status for “Physical fitness” and “Daily activities”, COPD status and the number of respiratory symptoms for “Social activities”, nationality and the number of respiratory symptoms for “Feelings”, sex and the number of respiratory symptoms for “Pain”, nationality for “Overall health”, and heating sources for “Change in health” (Table 5).

**Table 5:** Multiple-regression analyses of COOP/WONCA chart scores (dependent variables) and predictive variables

**Dependent variables**	**Predictive variables**	**β**	***P***	**R**^**2**^
				**Adjusted R**^**2**^
Physical fitness	Population group	0.125	< 0.001	
	Age	0.152	< 0.001	0.245
	Sex	0.132	< 0.001	0.240
	COPD status	0.252	< 0.001	
	Number of respiratory symptoms	0.161	< 0.001	
	Heating sources			
Feelings	Population group	−0.280	< 0.001	
	Age			0.121
	Sex			0.119
	COPD status			
	Number of respiratory symptoms	0.318	< 0.001	
	Heating sources			
Daily activities	Population group			
	Age	0.107	0.020	0.257
	Sex			0.254
	COPD status	0.396	< 0.001	
	Number of respiratory symptoms	0.184	< 0.001	
	Heating sources			
Social activities	Population group			
	Age	−0.114	0.002	0.204
	Sex			0.201
	COPD status	0.264	< 0.001	
	Number of respiratory symptoms	0.258	< 0.001	
	Heating sources			
Change of health	Population group	0.156	< 0.001	
	Age			0.045
	Sex			0.042
	COPD status			
	Number of respiratory symptoms			
	Heating sources	−0.204	< 0.001	
Overall health	Population group	0.216	< 0.001	
	Age			0.199
	Sex			0.195
	COPD status	0.197	< 0.001	
	Number of respiratory symptoms	0.172	< 0.001	
	Heating sources			
Pain	Population group	−0.185	< 0.001	
	Age			0.117
	Sex	0.207	< 0.001	0.112
	COPD status	0.129	0.005	
	Number of respiratory symptoms	0.234	< 0.001	
	Heating sources			

n = 737.

Population group (Japanese: 0, Chinese: 1) Sex (male: 0, female: 1), Heating sources (Biomass, coal, Animal dung: 1, Gas, Electric, Steam: 0) Number of respiratory symptoms (0–4), COPD status (health: 0, GOLD LV 1–4: 1–4).

### Comparison of health status between groups (nationality, sex, age, COPD status)

Using the results obtained from the stepwise multiple regression analyses; we compared the COOP/WONCA chart scores between the groups stratified by nationality, sex, age, and COPD status (moderate, severe). Differences in the scores between healthy subjects and patients with COPD were small in the Japanese group; health status was not affected by sex, age, or COPD status except for the group of the patients with moderate COPD aged 70–79 years (Table 6, upper panel). In contrast, in the Chinese group, differences were large between healthy subjects and patients with COPD, and the scores of all items except for “Change in health” tended to worsen in association with the progression of COPD in the Chinese group. There were no effects of sex or age between healthy subjects and the patients with moderate or severe COPD. Although a few scores were worse in older subjects and differed between the sexes, these results did not show any pattern.

**Table 6:** Comparison of COOP/WONCA chart scores between the Japanese and Chinese groups, stratified by race, sex, age, and COPD status

**Japanese**				**COOP/WONCA chart**						
**Group**	**Age (y)**	**Sex**	**n**	**Physical fitness**	**Feelings**	**Daily activities**	**Social activities**	**Change of health**	**Overall health**	**Pain**
Healthy subjects	50-59	Male	90	2.12 ± 0.92	2.21 ± 0.85	1.71 ± 0.84	1.52 ± 0.72	2.97 ± 0.32	2.78 ± 0.58	2.36 ± 0.88
		Female	77	2.86 ± 0.96	2.27 ± 0.77	1.70 ± 0.86	1.52 ± 0.58	2.90 ± 038	2.69 ± 0.65	2.68 ± 0.85
	60-69	Male	35	2.80 ± 1.05	2.26 ± 1.01	1.77 ± 0.97	1.40 ± 0.74	2.91 ± 0.37	2.51 ± 0.74	1.94 ± 0.80
		Female	27	3.07 ± 1.07	1.96 ± 0.85	1.70 ± 0.82	1.30 ± 0.82	2.96 ± 0.365	2.78 ± 0.70	2.33 ± 1.07
	70-79	Male	8	3.00 ± 1.20	2.13 ± 0.99	1.75 ± 0.71	1.75 ± 0.71	3.13 ± 0.35	3.13 ± 0.64	2.50 ± 0.53
		Female	6	2.17 ± 0.41	2.50 ± 1.52	1.50 ± 0.84	1.33 ± 0.52	3.00 ± 0.00	2.17 ± 0.75	2.50 ± 0.84
Patients with moderate COPD	50-59	Male	3	3.00 ± 0.00	2.00 ± 0.00	1.67 ± 1.15	1.00 ± 0.00	3.00 ± 0.00	3.00 ± 1.00	2.67 ± 0.58
		Female	4	2.50 ± 0.58	2.50 ± 1.29	1.50 ± 1.00	2.25 ± 1.50	3.25 ± 0.50	2.75 ± 1.26	3.25 ± 0.50
	60-69	Male	21	2.57 ± 0.93	1.76 ± 0.94	1.71 ± 1.01	1.38 ± 0.86	2.95 ± 0.50	2.81 ± 0.87	2.24 ± 1.00
		Female	15	2.93 ± 1.39	2.13 ± 0.92	1.87 ± 1.13	1.47 ± 1.13	2.67 ± 0.82	2.47 ± 0.83	2.40 ± 1.18
	70-79	Male	29	3.21 ± 1.50	1.83 ± 0.85	1.93 ± 1.03	1.17 ± 0.47	2.86 ± 0.83	2.76 ± 0.91	2.41 ± 1.24
		Female	17	2.82 ± 1.47	1.94 ± 0.90	1.41 ± 0.71	1.12 ± 0.33	2.00 ± 1.00	2.24 ± 0.83	2.82 ± 1.13
Patients with severe COPD	50-59	Male	1	3.00	3.00	2.00	3.00	3.00	3.00	4.00
		Female	0	-	-	-	-	-	-	-
	60-69	Male	8	3.38 ± 1.30	1.13 ± 0.35*	1.63 ± 1.19	1.00 ± 0.00	2.50 ± 0.93	2.63 ± 0.92	2.00 ± 1.20
		Female	3	3.00 ± 1.00	2.33 ± 1.15	2.00 ± 1.00	1.33 ± 0.58	3.33 ± 0.58	3.33 ± 0.58	3.00 ± 1.00
	70-79	Male	23	3.70 ± 1.36	1.87 ± 0.97	1.91 ± 1.12	1.30 ± 0.63	2.87 ± 0.76	2.83 ± 0.65	2.00 ± 1.04
		Female	3	3.33 ± 1.53	1.00 ± 0.00	1.33 ± 0.58	1.00 ± 0.00	2.33 ± 1.15	2.67 ± 0.58	1.67 ± 1.15
**Chinese**				**COOP/WONCA chart**						
**Group**	**Age (y)**	**Sex**	**n**	**Physical fitness**	**Feelings**	**Daily activities**	**Social activities**	**Change of health**	**Overall health**	**Pain**
Healthy subjects	50-59	Male	14	2.07 ± 1.27	1.21 ± 0.85	1.07 ± 0.27	1.00 ± 0.00	2.93 ± 0.27	2.43 ± 0.94	1.79 ± 1.12
		Female	51	2.51 ± 0.18	1.31 ± 0.47	1.20 ± 0.49	1.14 ± 0.53	3.02 ± 0.14	2.65 ± 1.00	1.78 ± 1.06
	60-69	Male	32	2.03 ± 1.12#	1.31 ± 0.69	1.13 ± 0.42	1.09 ± 0.39	2.94 ± 0.50	2.38 ± 0.91	1.41 ± 0.76
		Female	32	3.00 ± 1.37	1.44 ± 0.50	1.31 ± 0.64	1.19 ± 0.40	3.00 ± 0.51	2.88 ± 1.13	1.97 ± 1.06
	70-79	Male	32	3.03 ± 1.12	1.16 ± 0.45	1.03 ± 0.18	1.06 ± 0.25	2.94 ± 0.44	2.88 ± 0.83	1.59 ± 0.84#
		Female	38	3.74 ± 1.20	1.24 ± 0.43	1.13 ± 0.34	1.13 ± 0.34	3.05 ± 0.46	3.32 ±0.99	2.39 ±1.10
Patients with moderate COPD	50-59	Male	16	3.56 ± 0.81**	1.75 ± 0.86	2.19 ± 0.91	1.62 ± 0.72	2.56 ± 1.09	3.50 ± 0.63*	1.94 ± 0.93
		Female	18	3.44 ± 0.31	2.00 ± 0.97**	2.33 ± 0.84**	1.83 ± 0.71**	2.61 ± 0.98	3.44 ± 0.92	2.50 ± 1.04
	60-69	Male	21	3.52 ± 0.87**	1.90 ± 0.83	1.67 ± 0.66	1.76 ± 0.83	2.71 ± 1.10	3.19 ± 1.08*	2.33 ± 0.91*
		Female	17	3.41 ± 0.62	2.35 ± 0.49**	2.29 ± 0.69**	2.24 ± 0.66	3.18 ± 0.95	3.35± 0.61	2.35 ± 0.70
	70-79	Male	21	3.71 ± 0.78	2.14 ± 0.73**	2.24 ± 0.89**	2.00 ± 0.89*	3.19 ± 1.40	3.57± 0.75	2.10 ± 1.04
		Female	9	3.44 ± 0.88	2.33 ± 1.00**	2.33 ± 1.00**	2.22 ± 0.83**	2.78 ± 1.39	3.44± 0.73	2.78 ± 0.83
Patients with severe COPD	50-59	Male	16	4.06 ± 1.12**	2.13 ± 1.09	2.69 ± 1.25*	2.44 ± 1.36**	3.25 ± 1.00	3.56 ± 0.96*	2.50 ± 1.21
		Female	4	4.25 ± 0.65	3.25 ± 0.50**	4.00 ± 0.00**	4.00 ± 0.00**	4.00 ± 0.00**	4.00 ± 0.00	3.75 ± 0.50*
	60-69	Male	29	4.00 ± 1.10**	2.41 ± 1.12**	2.90 ±1.21**	2.79 ± 1.18**	3.14 ± 1.16	3.90 ± 0.77**	3.03 ± 1.12**
		Female	9	4.33 ± 0.71*	2.67 ± 1.32**	3.00 ± 1.41**	1.89 ± 1.05**	3.67 ± 0.71	4.22 ± 0.44**	3.22 ± 1.20*
	70-79	Male	27	4.26 ± 0.94**	2.52 ± 1.25**	3.19 ± 1.14**	2.78 ± 1.34**	3.30 ± 1.38	4.04 ± 0.81**	2.78 ± 1.09**#
		Female	14	4.50 ± 0.52	3.14 ± 1.10**	3.93 ± 1.14**	3.36 ± 1.35**	3.64 ± 1.01	3.86 ± 0.77	3.86 ± 0.86**

Data are expressed as mean ± SD.

*: P < 0.005, **: P < 0.001 vs. Healthy subjects.

# P < 0.005, Male vs. Female.

## Discussion

### Validity and reliability of the Japanese and Chinese versions of COOP/WONCA charts

The Chinese version of the COOP/WONCA charts [16] had already been shown to be reliable and valid for nervous system diseases, orthopedic disease, cancer, and strokes in China. Patients’ acceptance of the test is high, and the test is useful for the doctors who are evaluating them [16,23]. Although the Japanese version of the COOP/WONCA charts has been used to measure quality of life in the field of ophthalmology [24], and as a health survey and to measure quality of life in pediatrics [25,26], its reliability and validity have not been fully confirmed in an adult Japanese population. In our study, we enrolled subjects ages 50-79 years because the target of this study was patients with COPD, and the incidence of COPD increases with age. We verified the reliability and validity of the COOP/WONCA chart in Japanese and Chinese groups of healthy subjects and patients with COPD.

The “Feelings” and “Daily activities” COOP/WONCA charts showed high correlation with the mental component scores of the SF-36 in the Japanese population and the physical component scores of the SF-36 in the Chinese population. “Change in health”, which asks about changes in health status compared with that of the previous two weeks, was correlated with the physical component of SF-36 in both groups, but most with “physical functioning” in the Japanese group and most with “bodily pain” in the Chinese group. Other than these differences, the correlation of the COOP/WONCA charts and the subscales of SF-36 were consistent between the Japanese and Chinese groups. These results suggest that the Japanese and Chinese versions of the COOP/WONCA charts are appropriate for evaluating the health status of older healthy subjects and patients with COPD. The reproducibility of the COOP/WONCA charts, except for “Change in health”, was confirmed: in the test-retest, the ICC of each item was in the range of 0.657 to 0.890 in both the Japanese and Chinese populations.

We evaluated external validity and sensitivity by correlating the patient's evaluation with their doctor’s evaluation using the same COOP/WONCA charts, the number of respiratory symptoms associated with COPD, and the COPD status. After the subjects were matched on sex and age, the correlation coefficient between patients and their doctors was > 0.527 in the Japanese group, except for the “Pain” chart, and > 0.826 in the Chinese group for all of the items.

The Japanese patients with COPD gave higher scores on the “Pain” item than the doctor’s evaluation did, indicating that the doctor had underestimated the pain the patients felt. This discrepancy may be due in part to Japanese characteristics such as stoicism. The significant correlations of the COOP/WONCA chart items with the number of respiratory symptoms associated with COPD and with COPD status support the external validity of the charts. However, there were two aspects of the COOP/WONCA charts that differed between the Japanese and Chinese subjects. One was that there was no significant correlation between “Pain” and the number of respiratory symptoms of COPD in the Japanese group; the other was that all of the items, except “Change in health”, showed higher correlation with BI in the Chinese group than in the Japanese group. Pain control may have been more complete in Japanese patients with COPD than in Chinese patients. The reasons for the low correlation between BI and COOP/WONCA charts in the Japanese population are not clear. Cigarette type, smoking habits, and the tobacco smoking environment surrounding the patient may have contributed to these differences in the Japanese and Chinese groups.

To clarify the determinants of the COOP/WONCA chart items, we performed stepwise multiple regressions, adjusting by nationality, age, sex, COPD status, number of respiratory symptoms, and heating sources. The COPD status, the number of respiratory symptoms, or both were predictive variables for all of the items except “Change of health”. This result suggests that the health status measured by the COOP/WONCA charts reflected the levels and conditions of COPD patients. In addition, those scores on all items except “Feelings” and “Overall health” could be compared between the Japanese and Chinese populations according to the result from stepwise multiple regression analyses. Although nationality was a predictive variable for “Feelings” and “Overall health”, the scores for “Feelings” among healthy subjects were better in the Chinese group than in the Japanese group, whereas for the COPD patients, the “Overall health” score was better in the Japanese group than in the Chinese group. The social environment surrounding the patients, lifestyle, cultural and religious background, and medical treatments may influence “Feelings” and “Overall health” in complicated ways. It is necessary to analyze this point in more detail. The reliability and validity of the English version of the COOP/WONCA charts has been tested with COPD patients. Stavem and Jodalen [19] observed high correlation between items on the COOP/WONCA charts and the EuroQol 5-Dimension (EQ-5D) test in 59 male and female outpatients with COPD (average age 57 years old). The authors suggested that the reliability of the COOP/WONCA items was acceptable for use at the group level, but lower than current recommendations for use in individual patients. In contrast, Eaton et al. [17] found that the Dartmouth COOP charts were reliable, valid, and responsive compared with the SF-36, Chronic Respiratory Questionnaire (CRQ), and Hospital Anxiety and Depression (HAD) questionnaires in a comparative study of oxygen therapy for patients with COPD, who were on average 68.3 years old. The authors showed that these charts are a simple, reliable health-related quality-of-life tool that was valid and responsive in their population of COPD patients and may have a valuable role in routine clinical practice. Our results also suggested that the Japanese and Chinese versions of the COOP/WONCA charts provide good reliability and validity for measuring health status and for comparing healthy subjects and patients with COPD in Japanese and Chinese populations.

### Comparison of health status in Japanese and Chinese patients with COPD by using COOP/WONCA charts

1. In our Japanese subjects, patients with COPD showed almost the same health status as healthy subjects of the same age, when stratified by sex, age, and COPD status (Table 6). However, although the healthy Chinese subjects had clearly higher health status than the Japanese subjects, the health status declined in proportion to GOLD stage in patients with COPD. In particular, for Chinese subjects, the physical score and daily and social activity scores were worse in the patients with moderate COPD than in the healthy subjects, and the pain and psychological health status worsened further in the patients with severe COPD. The poor “Overall health” in the patients with severe COPD may reflect the strong deterioration of physical and social activity and psychological performance.

Few studies have used the COOP/WONCA charts to evaluate chronic disease and health status in COPD patients and have compared the results between countries. There is only one report that we know of that compared the results of a factor analysis of COOP/WONCA charts at primary care clinics in four countries and showed the average scores on each item and the 95% confidence interval [20]. The authors suggested that the COOP/WONCA system was suitable for general use in primary care internationally.

The present study clarified that although healthy Chinese 50- to 79-year-olds have higher health status than corresponding Japanese people, the health status of Chinese patients with COPD decreases as symptoms get worse. Because a high positive correlation was observed between indoor use of smoky fuels and “Daily activity” and “Social activity” scores in the Chinese group, use of these fuels may be one of the precipitating factors of COPD in the Chinese population. More than 20% of Chinese males and females in this study were using coal or biomass indoors; indoor use of smoky fuels was associated with worse respiratory symptoms, which restrained physical and social activity. Diette et al. [27] reviewed the relation of biomass combustion exposure in an indoor environment and pulmonary obstructive disease, and found that combustion of biomass or coal indoors can lead to a fall in pulmonary function and aggravation of COPD. Therefore, the use of indoor heating and cooking equipment that does not produce smoke and the avoidance of cigarette smoking may improve physical and social activity and psychological levels as well as medical treatment, including pain control. Future studies should analyze risk factors related to health status in patients with COPD in the Chinese population. Aging and air pollution are increasing rapidly in Asia, which will likely increase the rate of COPD disease and related mortality. Immediate action to reduce risk factors leading to COPD may be required for improving the health economic and social burdens caused by the disability-adjusted life years lost due to COPD.

## Conclusions

The Japanese and Chinese versions of the COOP/WONCA charts showed good reliability and validity for measuring health status and for comparing healthy subjects and patients with COPD in Japanese and Chinese populations.

The physical, psychosocial activities, and pain scores increased significantly as COPD status worsened in Chinese subjects, whereas these scores were not affected by sex, age, or COPD status for Japanese subjects. Brinkman index and indoor use of smoky fuel affected the COOP/WONCA scores in Chinese patients but not in Japanese patients. These results suggest that countermeasures against insufficient health care and smoky environments may improve the health status of Chinese patients with COPD.

## Abbreviations

COPD: Chronic obstructive pulmonary disease; WONCA: World Organization of National Colleges, Academies and Academic Associations of General Practitioners/Family Physicians; SF-36: SF-36v2™; GOLD: Global initiative for chronic obstructive lung disease; BI: Brinkman index; ICC: Intraclass correlation coefficient; CRQ: Chronic Respiratory Questionnaire; HAD questionnaire: Hospital Anxiety and Depression questionnaire.

## Competing interests

Authors have no competing interests to declare.

## Authors' contributions

MY was a graduate student at Kurume University and was involved in the literature search, data collection, and data editing. She took the lead in developing this manuscript. MN supervised MY; was involved in data editing, analysis and interpretation; provided discussion and advice; and helped to prepare the manuscript. H Obata was an advisor and was actively involved in data collection in Yamaguchi prefecture. HI and TK were also advisors and were actively involved in data collection in Nagasaki prefecture. QW was actively involved in data collection in the People’s Republic of China. YH was an advisor and was involved in the literature search and data collection. H Omori was actively involved in data collection in Kumamoto prefecture. YI supervised MY and was involved in the literature search; data collection, editing, analysis, and interpretation; provided discussion and advice; helped with manuscript preparation; and participated in the development of the study proposal. She also supervised all aspects of this study. All authors read and approved the final manuscript.

## Pre-publication history

The pre-publication history for this paper can be accessed here:

http://www.biomedcentral.com/1471-2458/13/754/prepub

## Supplementary Material

Additional file 1 SF-36v2 questionnaire for health survey. Click here for file

Additional file 2 The Dartmouth COOP Functional Health Assessment Charts/WONCA (The COOP/WONCA Charts). Click here for file
