# Supplementary material for: Application of the COOP/WONCA charts to aged patients with chronic obstructive pulmonary disease: a comparison between Japanese and Chinese populations
Source: BMC Public Health. 2013 Aug 15;13:754. doi: 10.1186/1471-2458-13-754 (PMC3765517; doi:10.1186/1471-2458-13-754)
Supplement: Additional file 1 — SF-36v2 questionnaire for health survey. [file 1471-2458-13-754-S1.pdf]

The SF-36v2™ Health Survey

Instructions for Completing the Questionnaire

Please answer every question. Some questions may look like others, but each one is different. Please take the time to read and answer each question carefully by filling in the bubble that best represents your response.

EXAMPLE

This is for your review. Do not answer this question. The questionnaire begins with the section ***Your Health in General*** below.

For each question you will be asked to fill in a bubble in each line:

1. **How strongly do you agree or disagree with each of the following statements?**

	Strongly agree	Agree	Uncertain	Disagree	Strongly disagree
a) I enjoy listening to music.	○	●	○	○	○
b) I enjoy reading magazines.	●	○	○	○	○

Please begin answering the questions now.

Your Health in General

1. **In general, would you say your health is:**

Excellent	Very good	Good	Fair	Poor	
○1	○2	○3	○4	○5	GH01

2. **Compared to one year ago, how would you rate your health in general now?**

Much better now than one year ago	Somewhat better now than one year ago	About the same as one year ago	Somewhat worse now than one year ago	Much worse now than one year ago	
○1	○2	○3	○4	○5	HT

Please turn the page and continue.

3. **The following questions are about activities you might do during a typical day. Does <u>your health now limit you</u> in these activities? If so, how much?**

	Yes, limited a lot	Yes, limited a little	No, not limited at all	
a) **Vigorous activities**, such as running, lifting heavy objects, participating in strenuous sports	○1	○2	○3	PF01
b) **Moderate activities**, such as moving a table, pushing a vacuum cleaner, bowling, or playing golf	○1	○2	○3	PF02
c) Lifting or carrying groceries	○1	○2	○3	PF03
d) Climbing **several** flights of stairs	○1	○2	○3	PF04
e) Climbing **one** flight of stairs	○1	○2	○3	PF05
f) Bending, kneeling, or stooping	○1	○2	○3	PF06
g) Walking **more than a mile**	○1	○2	○3	PF07
h) Walking **several hundred yards**	○1	○2	○3	PF08
i) Walking **one hundred yards**	○1	○2	○3	PF09
j) Bathing or dressing yourself	○1	○2	○3	PF10

4. **During the <u>past 4 weeks</u>, how much of the time have you had any of the following problems with your work or other regular daily activities <u>as a result of your physical health</u>?**

	All of the time	Most of the time	Some of the time	A little of the time	None of the time	
a) Cut down on the **amount of time** you spent on work or other activities	○1	○2	○3	○4	○5	RP01
b) **Accomplished less** than you would like	○1	○2	○3	○4	○5	RP02
c) Were limited in the **kind** of work or other activities	○1	○2	○3	○4	○5	RP03
d) Had **difficulty** performing the work or other activities (for example, it took extra effort)	○1	○2	○3	○4	○5	RP04

5. **During the past 4 weeks, how much of the time have you had any of the following problems with your work or other regular daily activities as a result of any emotional problems (such as feeling depressed or anxious)?**

	All of the time	Most of the time	Some of the time	A little of the time	None of the time	
a) Cut down on the **amount of time** you spent on work or other activities	O1	O2	O3	O4	O5	RE01
b) **Accomplished less** than you would like	O1	O2	O3	O4	O5	RE02
c) Did work or other activities **less carefully than usual**	O1	O2	O3	O4	O5	RE03

6. **During the past 4 weeks, to what extent has your physical health or emotional problems interfered with your normal social activities with family, friends, neighbors, or groups?**

Not at all	Slightly	Moderately	Quite a bit	Extremely	
O1	O2	O3	O4	O5	SF01

7. **How much bodily pain have you had during the past 4 weeks?**

None	Very mild	Mild	Moderate	Severe	Very severe	
O1	O2	O3	O4	O5	O6	BP01

8. **During the past 4 weeks, how much did pain interfere with your normal work (including both work outside the home and housework)?**

Not at all	A little bit	Moderately	Quite a bit	Extremely	
O1	O2	O3	O4	O5	BP02

9. **These questions are about how you feel and how things have been with you during the past 4 weeks. For each question, please give the one answer that comes closest to the way you have been feeling. How much of the time during the past 4 weeks...**

	All of the time	Most of the time	Some of the time	A little of the time	None of the time	
a) did you feel full of life?	O1	O2	O3	O4	O5	VT01
b) have you been very nervous?	O1	O2	O3	O4	O5	MH01
c) have you felt so down in the dumps that nothing could cheer you up?	O1	O2	O3	O4	O5	MH02
d) have you felt calm and peaceful?	O1	O2	O3	O4	O5	MH03
e) did you have a lot of energy?	O1	O2	O3	O4	O5	VT02
f) have you felt downhearted and depressed?	O1	O2	O3	O4	O5	MH04
g) did you feel worn out?	O1	O2	O3	O4	O5	VT03
h) have you been happy?	O1	O2	O3	O4	O5	MH05
i) did you feel tired?	O1	O2	O3	O4	O5	VT04

10. **During the <u>past 4 weeks</u>, how much of the time has your <u>physical health or emotional problems</u> interfered with your social activities (like visiting friends, relatives, etc.)?**

All of the time	Most of the time	Some of the time	A little of the time	None of the time	
O1	O2	O3	O4	O5	SF02

11. **How TRUE or FALSE is <u>each</u> of the following statements for you?**

	Definitely true	Mostly true	Don't know	Mostly false	Definitely false	
a) I seem to get sick a little easier than other people	O1	O2	O3	O4	O5	GH02
b) I am as healthy as anybody I know	O1	O2	O3	O4	O5	GH03
c) I expect my health to get worse	O1	O2	O3	O4	O5	GH04
d) My health is excellent	O1	O2	O3	O4	O5	GH05

SF-36v2™ - © 1999 – QualityMetric, Inc. – All Rights Reserved
