# Supplementary material for: Application of the COOP/WONCA charts to aged patients with chronic obstructive pulmonary disease: a comparison between Japanese and Chinese populations
Source: BMC Public Health. 2013 Aug 15;13:754. doi: 10.1186/1471-2458-13-754 (PMC3765517; doi:10.1186/1471-2458-13-754)
Supplement: Additional file 2 — The Dartmouth COOP Functional Health Assessment Charts/WONCA (The COOP/WONCA Charts). [file 1471-2458-13-754-S2.pdf]

The Dartmouth COOP Functional Health Assessment Charts/WONCA (The COOP/WONCA Charts)

The COOP/WONCA charts may be used for research and clinical care. Permission to use the COOP/WONCA charts specifically exclude the right to distribute, reproduce or share them in any form for commercial purposes.

Copyright © Trustee of Dartmouth College/COOP Project 1989 Support provided by the Henry J Kaiser Family Foundation

The available versions of the COOP/WONCA charts

English

Physical fitness

During the past 2 weeks...
What was the hardest physical activity you could do for at least 2 minutes?

**Very heavy, (for example) run, at a fast pace**	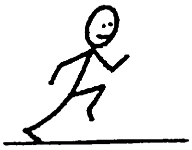	**1**
**Heavy, (for example) jog, at a slow pace**	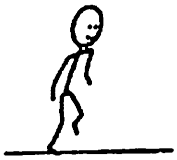	**2**
**Moderate, (for example) walk, at a fast pace**	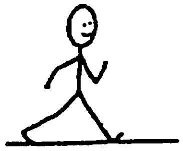	**3**
**Light, (for example) walk, at a medium pace**	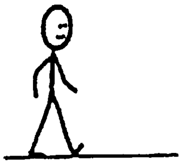	**4**
**Very light, (for example) walk, at a slow pace or not able to walk**	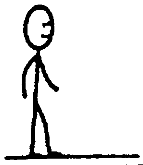	**5**

DARTMOUTH COOP FUNCTIONAL ASSESSMENT CHARTS/WONCA
COPYRIGHT© TRUSTEES OF DARTMOUTH/COOP PROJECT 1995

Feelings

During the past 2 weeks...
How much have you been bothered by emotional problems such as feeling anxious, depressed, irritable or downhearted and sad?

Not at all	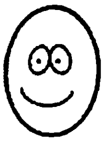 1
Slightly	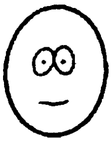 2
Moderately	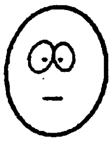 3
Quite a bit	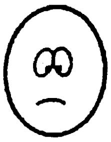 4
Extremely	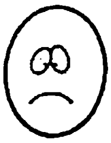 5

DARTMOUTH COOP FUNCTIONAL ASSESSMENT CHARTS/WONCA
COPYRIGHT©TRUSTEES OF DARTMOUTH/COOP PROJECT 1995

Daily activities

During the past 2 weeks...
How much difficulty have you had doing your usual activities or tasks, both inside and outside the house because of your physical and emotional health?

No difficulty at all	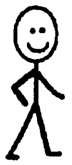	1
A little bit of difficulty	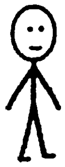	2
Some difficulty	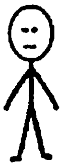	3
Much difficulty	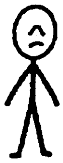	4
Could not do	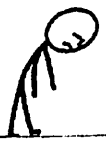	5

DARTMOUTH COOP FUNCTIONAL ASSESSMENT CHARTS/WONCA
COPYRIGHT©TRUSTEES OF DARTMOUTH/COOP PROJECT 1995

Social activities

During the past 2 weeks...
Has your physical an emotional health limited your social acivities with family, friends, neigbours or groups?

Not at all	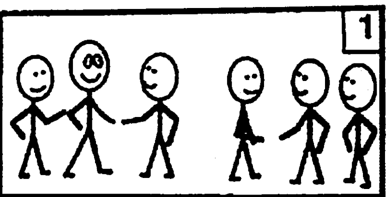	1
Slightly	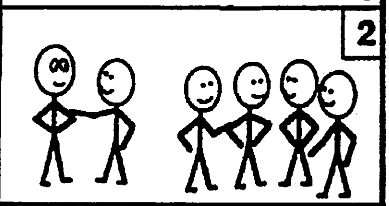	2
Modorately	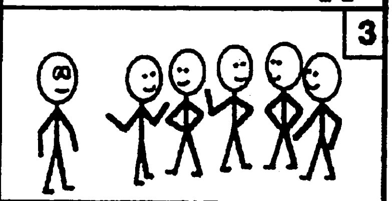	3
Quite a bit	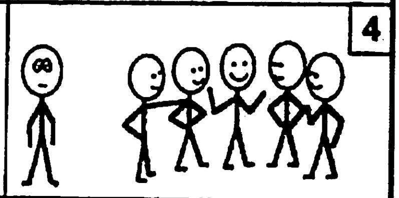	4
Extremely	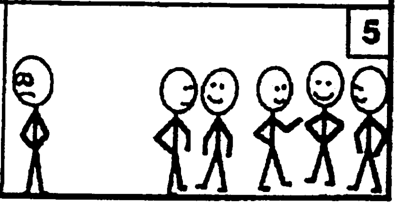	5

DARTMOUTH COOP FUNCTIONAL ASSESSMENT CHARTS/WONCA
COPYRIGHT©TRUSTEES OF DARTMOUTH/COOP PROJECT 1995

Change in health

How would you rate your overall health now compared to 2 weeks ago?

**Much better**	⬆⬆ ++	1
**A little better**	⬆ +	2
**About the same**	⬅➡ =	3
**A little worse**	⬇ −	4
**Much worse**	⬇⬇ −−	5

DARTMOUTH COOP FUNCTIONAL ASSESSMENT CHARTS/WONCA
COPYRIGHT© TRUSTEES OF DARTMOUTH/COOP PROJECT 1995

Overall health

During the past 2 weeks...
How would you rate your health in general?

**Excellent**	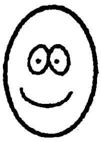	1
**Very good**	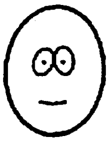	2
**Good**	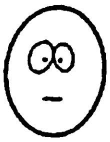	3
**Fair**	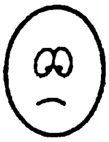	4
**Poor**	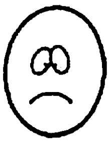	5

DARTMOUTH COOP FUNCTIONAL ASSESSMENT CHARTS/WONCA
COPYRIGHT©TRUSTEES OF DARTMOUTH/COOP PROJECT 1995

PAIN

During the past 4 weeks . . .
How much bodily pain have you generally had?

No pain	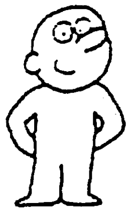	1
Very mild pain	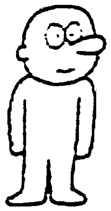	2
Mild pain	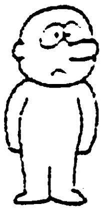	3
Moderate pain	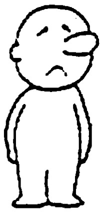	4
Severe pain	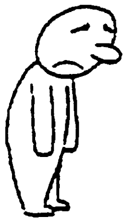	5

Dartmouth COOP Functional Health Assessment Charts/WONCA
Copyright © Trustees of Dartmouth College/COOP Project 1989
Support Provided by The Henry J. Kaiser Family Foundation
